# Testing the effects of 4-week training programs based on extreme and medium-sided soccer games: a study focusing on change-of-direction, vertical jump height and locomotor profile

**DOI:** 10.1186/s13102-022-00592-1

**Published:** 2022-11-24

**Authors:** Piotr Makar, Gibson Praça, Adam Kawczyński, Zeki Akyildiz, Mehmet yıldız, Rodrigo Aquino, Filipe Manuel Clemente

**Affiliations:** 1 Gdańsk University of Physical Education and Sport, Gdańsk, Poland; 2 Sports Department, Universidade Federal de Minas Gerais, Belo Horizonte, Brazil; 3 Faculty of Sport Sciences, Gazi University, 06560 Ankara, Turkey; 4 Sports Science Faculty, Afyon Kocatepe University, Afyonkarahisar, Turkey; 5 LabSport, Department of Sports, Center of Physical Education and Sports, Federal University of Espírito Santo, Vitória, 29075-910 Brazil; 6 Escola Superior Desporto e Lazer, Instituto Politécnico de Viana do Castelo, Rua Escola Industrial e Comercial de Nun’Álvares, 4900-347 Viana Do Castelo, Portugal; 7 Research Center in Sports Performance, Recreation, Innovation and Technology (SPRINT), 4960-320 Melgaço, Portugal; 8 Instituto de Telecomunicações, Delegação da Covilhã, 1049-001 Lisbon, Portugal

**Keywords:** Football, Athletic performance, Constrained games, Exercise testing

## Abstract

**Aim:**

This study tested the effects of two training programs (one program based on extreme sided-games of 1v1 vs. one program based on the medium-sided game of 5v5) on the physical fitness adaptations of youth soccer players. In specific, it was analyzed the effects of the training programs on the 5–0–5 change-of-direction time (5–0–5 time), countermovement jump (CMJ), and final velocity in the 30–15 Intermittent Fitness test (VIFT).

**Methods:**

This study followed a randomized parallel study design. Twenty male regional-level soccer players (age: 17.0 ± 0.3 years old) were randomly assigned to two groups: (1) the 1v1 format; and (2) the 5v5 format. The training intervention lasted four weeks. The week before (baseline) and the week after the intervention, the participants were assessed in the 5–0–5 change-of-direction test (measured using timing gates), CMJ (measured by photoelectric cells), and 30–15 Intermittent Fitness test. The training intervention consisted of 8 sessions (2 sessions per week). The 1v1 group performed four repetitions of 30 s in each session, while the 5v5 group performed four repetitions of 4 min.

**Results:**

The 5–0–5 time changed − 4.82% (*p* = 0.004; d = 1.115) for the 1v1 group and − 4.26% (*p* = 0.004; d = 0.859) for the 5v5 group. CMJ changes occurred both in the 1v1 and 5v5 group and amounted to 1.7% (*p* = 0.003; d = 0.509) and 1.2% (*p* = 0.263; d = 0.155) respectively. VIFT changed 2.6% (*p* = 0.718; d = 0.178) for the 1v1 group and 3.0% (*p* = 0.593; d = 0.274) for the 5v5 group. The 1v1 group reported significantly lower post-intervention 5–0–5 time than the 5v5 group (− 4.3%; *p* = 0.048; d = 0.954), although no significant differences in CMJ (3.2%; *p* = 0.147; d = 0.678) and VIFT (2.5%; *p* = 0.697; d = 0.177) were revealed.

**Conclusions:**

The extreme-sided games meaningfully beneficiated the vertical jump height and change-of-direction performance of youth soccer players. The extreme-sided games seem more beneficial than medium-sided games for improving these physical abilities while showing that four weeks were enough to impact the players significantly.

**Supplementary Information:**

The online version contains supplementary material available at 10.1186/s13102-022-00592-1.

## Introduction

Small-sided games are constrained game-based drills often used to simulate the official game's dynamics while exercising players based on a specific goal projected by the coach [1]. These games are popular in soccer practice and science mainly because coaches and sports scientists design them to develop specific physiological, physical, technical, and tactical aspects [2]. There is a myriad of task constraints used when creating the small-sided game, such as a format (e.g., extreme—1 vs. 1; small—2 vs. 1 to 4 vs. 4; medium—5 vs. 4 to 7 vs. 7; large > 8 vs. 7) [3], a pitch size based on a relative area per player (e.g., small—50 m^2^ per player; medium—100 m^2^ per player; large—250 m^2^ per player) [4], and rules (e.g., scoring methods, action restriction) [5].

The analysis of acute effects related to the implementation of SSGs shows that the number of players (format) involved significantly affects the physical and physiological responses [6]. For example, it has been consistently observed that extreme-sided games (e.g., 1v1) or smaller formats (e.g., 2v2–4v4) are more intense than remaining formats (5v5 or greater) in regards to heart rate or rate of perceived exertion [2]. On the other hand, larger formats (for example 6v6 and 8v8) are more demanding in the distance covered above 14 km/h, while smaller formats (for example 4v4) are highly demanding for mechanical work [7]. This can be explained by the fact that larger play formats also require a greater field dimension, promoting interaction between factors. Considering the relationship with pitch dimensions, it is observed that larger pitches enhance the distance covered at high-speed running and also contribute to the intensification of heart rate responses [8]. This fact can be justified by the change in the dynamic of the play and the availability to explore the space while reaching greater intensities of running.

Few studies have investigated the effects of different small-sided game formats on long-term and chronic physical and physiological adaptations [2]. The studies on this topic usually compared the impact of small-sided games and running-based high-intensity interval training (HIIT) on aerobic and neuromuscular adaptations [9]. Consistent results showed that small-sided games improve aerobic performance in field-based tests [10]. However, there are also some inconsistent findings revealing the usefulness of small-sided games for improving jump performance, linear sprint, repeated sprint ability, and change of direction [5]. Therefore, further studies are necessary to investigate the effects of different small-sided game formats on long-term neuromuscular adaptations.

Running-based HIIT involves repeated short (< 45 s of high but not all-out intensity exercise) to long (2–4 min of high, not maximal intensity exercise) bouts [11]. Buchheit and Laursen [11, 12] suggested that the short HIIT format provides greater acute central demands than the long HIIT format. However, both HIIT types promote similar acute peripheral responses. Therefore, different formats of small-sided games (e.g., extreme [short] vs. medium [long])—a type of HIIT [13]—can provide various physical adaptations in soccer players.

It is vital to analyze how different SSGs can impact the physical adaptations of soccer players. Considering that other play formats significantly impact players' acute responses, it is expected to observe differences in long-term adaptations. However, this hypothesis is still an uncovered area. In the particular case of youth populations competing at the regional level, this is essential since most of the training practice requires new information from research to help coaches to make better decisions since they do not often have monitoring instruments to predict the curve of adaptations. Based on this approach, this study aimed to test the effects of two training programs (one program based on extreme sided-games of 1v1 vs. one program based on the medium-sided game of 5v5) on the physical fitness adaptations of youth soccer players. Specifically, the effects of the training programs on the 5–0–5 time, countermovement jump (CMJ), and final velocity in the 30–15 Intermittent Fitness test (VIFT) were analyzed.

## Methods

### Study design

This study followed a simple randomized parallel study design. The Research Randomizer software was used to run the process. The ethical committee of the University of Gazi University, Ankara, Turkey, approved the protocol with the code number (2021/1166, authorized on 27.12.2021). Twenty players were randomly allocated to two groups (1v1 and 5v5), for which small-sided games supplemented regular training sessions. The SSGs training formats were selected based on previous research, which suggests significant differences in physiological and locomotor intensities coming from extreme-sided games such as 1 versus 1 and 5 versus 5 medium-sided games [14, 15]. Simple randomization was performed after the first physical fitness assessment. Baseline differences do not reveal significant differences between the groups.

### Setting and context

The whole study started on May 09, 2022 (baseline physical fitness assessments) and ended on June 05, 2022 (post-intervention physical fitness assessment). The timeline of the study is presented in Table 1. The players participated in a 4-week training intervention. The time of the study was associated with the convenience period found for introducing the contents in the team's schedule. In the first week, the participants were assessed for change-of-direction, countermovement jump, and 30–15 Intermittent Fitness test. For four weeks after the assessment, they participated in the dedicated training intervention sessions (two per week), and in the sixth-week physical fitness, assessments were repeated. The physical fitness assessments performed at baseline and post-intervention preceded a 24-h rest period. The assessments were carried out at 5 pm, with environmental conditions of 23 °C and 65% relative humidity at baseline assessment and 16 °C and relative humidity of 68% at the post-intervention. During the 4-week training period, the average temperature of sessions was 20.5 ± 4.9 °C and 61.3 ± 8.1% relative humidity.

**Table 1:** Timeline of the study

May 09	May 11, 2022	May 13, 2022	May 17, 2022	May 19, 2022	May 25, 2022	May 27, 2022	June 01, 2022	June 03, 2022	June 05
Baseline	Session 1	Session 2	Session 3	Session 4	Session 5	Session 6	Session 7	Session 8	Pos-intervention
Physical fitness assessment	Group A performed 4 repetitions of 30 s (interspaced by 2 min rest) while playing 1v1 format	Group A performed 4 repetitions of 30 s (interspaced by 2 min rest) while playing 1v1 format	Group A performed 4 repetitions of 30 s (interspaced by 2 min rest) while playing 1v1 format	Group A performed 4 repetitions of 30 s (interspaced by 2 min rest) while playing 1v1 format	Group A performed 4 repetitions of 30 s (interspaced by 2 min rest) while playing 1v1 format	Group A performed 4 repetitions of 30 s (interspaced by 2 min rest) while playing 1v1 format	Group A performed 4 repetitions of 30 s (interspaced by 2 min rest) while playing 1v1 format	Group A performed 4 repetitions of 30 s (interspaced by 2 min rest) while playing 1v1 format	Physical fitness assessment
	Group B performed 4 repetitions of 4 min (interspaced by 2 min rest) while playing 5v5 format	Group B performed 4 repetitions of 4 min (interspaced by 2 min rest) while playing 5v5 format	Group B performed 4 repetitions of 4 min (interspaced by 2 min rest) while playing 5v5 format	Group B performed 4 repetitions of 4 min (interspaced by 2 min rest) while playing 5v5 format	Group B performed 4 repetitions of 4 min (interspaced by 2 min rest) while playing 5v5 format	Group B performed 4 repetitions of 4 min (interspaced by 2 min rest) while playing 5v5 format	Group B performed 4 repetitions of 4 min (interspaced by 2 min rest) while playing 5v5 format	Group B performed 4 repetitions of 4 min (interspaced by 2 min rest) while playing 5v5 format	

### Participants

The G*power (version 3.1.9.6.) software was used to estimate the sample size. The recommended sample size was n = 20, for a power of 0.80, an effect size of 0.33, and a p of 0.05. A convenience sampling strategy was used to select the players. The inclusion criteria were as follows: (1) outfield players; (2) players who participated in all training sessions on the days of data collection (8 training sessions over four consecutive weeks); (3) players who did not take any drugs, or energy drinks, or changed any of the daily dietary routines. Out of a total number of twenty-three possible participants (all belong to the same team), twenty were selected.

Three were excluded based on playing their goalkeeper position. The twenty participants presented a training adherence of 100%. The absence in training and assessment sessions was not reported during the four weeks. The twenty male youth soccer players (age: 17 years old; 7.5 ± 1.5 years of experience; 176.0 ± 5.3 cm of stature; 67.4 ± 4.9 kg of body mass) belong to the same team competing at the national level. The team had an average of 4 training sessions a week, plus an official match at weekends. The training sessions lasted between 90 to 100 min. The twenty players were randomly assigned (simply a randomization process) to two groups after the baseline assessments. Group A (n = 10) was exposed to the 1v1 format twice a week over four weeks, while group B (n = 10) was to the 5v5 format twice a week over four weeks. The participants and their legal guardians received a letter and verbal instructions about the study design, risks, and benefits. Once the participants agreed to participate in the study, they voluntarily signed a free consent form. The study followed the ethical standards for the study of humans as described in the Declaration of Helsinki.

### Training intervention

The two groups shared regular training sessions and official matches with the team. They only differ from the team players' participation in the additional intervention. Twice a week, the participants were enrolled in the 1v1 (group A) and 5v5 (group B) formats of play immediately after completing the FIFA11 + warm-up protocol [16]. Group A performed the 1v1 format in a 15 × 10 m field (length: width ratio of 1.5; area per player of 75 m^2^), with a small goal (2 × 2 m) centered in the endline. The 1v1 format was performed in 4 repetitions of 30 s, interspaced by 2 min of passive rest between them. Group B performed the 5v5 format in a 40 × 30 m field (length: width ratio of 1.3; area per player of 120 m^2^), with a small goal (2 × 2 m) centered in the endline. The 5v5 format was performed in 4 repetitions of 4 min, along with a two-minute-passive rest period between them. Goalkeepers were not used in both formats. No verbal encouragement was provided. No offside rule was used, and the ball was repositioned with the foot. The other soccer rules were applied. Within the group, the coach assigned the players to ensure skill-proficiency balance. Both teams and opponents were always the same.

### Procedures of physical fitness assessment

The physical fitness assessment was conducted the week before and the week after the 4-week training intervention. The assessments were preceded by a 24 h rest period after the last training session. The assessments took place on synthetic turf at 5 pm. The players had slept 6.4 ± 1.5 h the night before the evaluations and had an average score of 5.9 ± 1.7 arbitrary units on the total quality recovery scale. Both assessments were carried out at the same day of the week under similar conditions. The assessments started with a standardized warm-up protocol (FIFA11 + warm-up protocol) [16] followed by a 3-min rest period. After that, the players performed the CMJ, the 5–0–5 change of direction test, and the 30–15 Intermittent Fitness test. The testes were interspaced by a 3-min rest period.

### Countermovement jump test

The CMJ test with hands on the hips was implemented as described in previous protocols [17]. The Optojump photoelectric cells (Microgate, Bolzano, Italy) consisting of two parallel bars were placed approximately 1 m apart and parallel to each other. The validity and reliability of Optojump photoelectric cells to measure the vertical jump height had been previously confirmed [18]. The participants performed three trials of CMJ, interspaced by a 30-s rest period. The average of the within-player coefficient of variation across the trials conducted at baseline was 1.9% and at post-intervention assessment was 3.0%. The best score (the highest jump) obtained in each assessment moment was used for further data treatment.

### 5–0–5 change of direction test

The traditional 5–0–5 change of direction test was implemented as described in previous protocols [19]. The time at the test was measured from a dual-beam timing gate system (Smart Speed, Fusion Sport, Queensland, Australia), with all times recorded to the nearest 0.01 s. The players started with the preferred leg at the front, and change-of-direction performance always with the same preferred leg. The players began the test at a fixed line of 0.3 m behind the first pair of photocells. The players performed two trials of the 5–0–5 test, interspaced by a 3-min rest period. The average of the within-player coefficient of variation across trials conducted at baseline was 4.1%, and during post-intervention assessment amounted to 4.4%. The best score (fastest time) obtained in each assessment moment was used for further data treatment.

### The 30–15 intermittent fitness test

The 30–15 Intermittent Fitness Test was implemented following the original protocol [20]. The tests consist of 30-s shuttle runs interspaced by 15 s of recovery. The test starts at 8 km/h and progressively increases by 0.5 km/h in each new 30-s work period. An audio beep governed the player’s pace. All participants were familiar with the test. The test ended when a player was exhausted or failed to reach the determined line twice. The final velocity ultimately attained was obtained from the last completed stage. The final velocity at 30–15 Intermittent Fitness Test was used as the primary outcome.

### Statistical procedures

Descriptive statistics are presented in the form of the mean and standard deviation. The normality and homogeneity of the sample were preliminarily inspected using Shapiro–Wilk and Levene’s tests, respectively. Once the assumptions of normality (*p* > 0.05) and homogeneity (*p* > 0.05) of the sample were confirmed, a mixed ANOVA (time*group) was executed to analyze the variations of physical fitness assessments after the intervention period. Partial eta squared (${\eta }_{p}^{2}$) was used as the effect size technique. The Bonferroni test was used as posthoc tests. The standardized effect size of Cohen (d) was adopted to estimate the effect size for pairwise comparisons. The statistical procedures were executed in the SPSS software (version 28.0.0.0, IBM, Chicago, USA) for a *p* < 0.05.

## Results

The mixed ANOVA revealed no significant interactions (time–group) between the 5–0–5 time (F = 0.053; *p* = 0.820; ${\eta }_{p}^{2}$=0.003), CMJ (F = 0.297; *p* = 0.592; ${\eta }_{p}^{2}$=0.016) and VIFT (F = 0.003; *p* = 0.954; ${\eta }_{p}^{2}$<0.001).

**Fig. 1:**
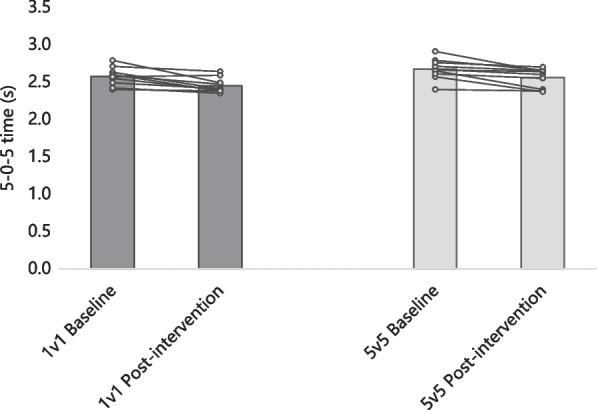
Group average and intra-individual variation of 5–0–5 time

Figure 1 presents the group average and the intra-individual variation of 5–0–5 times. The 5–0–5 time changed − 4.82% (2.6 ± 0.1 s baseline to 2.5 ± 0.1 s post-intervention; *p* = 0.004; d = 1.115) for the 1v1 group and − 4.26% (2.7 ± 0.1 s baseline to 2.6 ± 0.1 s post-intervention; *p* = 0.004; d = 0.859) for the 5v5 group. No significant differences were found between the groups in the 5–0–5 time at the baseline (3.8% difference; *p* = 0.107; d = 0.760), although the 1v1 group reported significantly lower post-intervention 5–0–5 time (–4.3%; *p* = 0.048; d = 0.954).

**Fig. 2:**
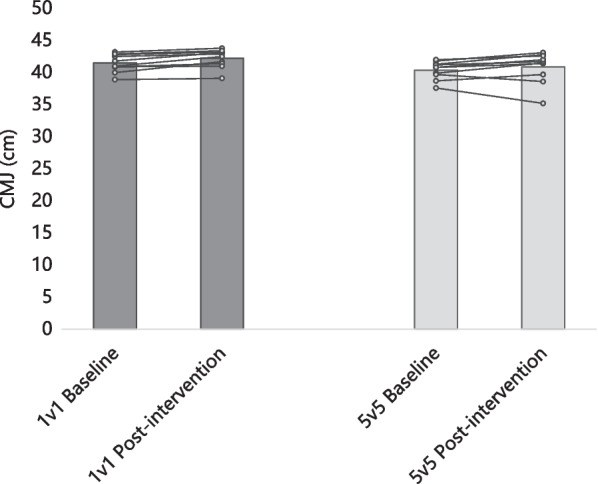
Group average and intra-individual variation of CMJ (countermovement jump test)

Figure 2 shows the group average and the intra-individual variation of CMJ. CMJ changed 1.7% (41.5 ± 1.4 cm baseline to 42.2 ± 1.4 cm post-intervention; *p* = 0.003; d = 0.509) for the 1v1 group and 1.2% (40.4 ± 1.4 cm baseline to 40.9 ± 2.4 cm post-intervention; *p* = 0.263; d = 0.155) for the 5v5 group. No significant differences were found between the groups in the CMJ at the baseline (2.7% difference; *p* = 0.094; d = 0.792) and post-intervention (3.2%; *p* = 0.147; d = 0.678).

**Fig. 3:**
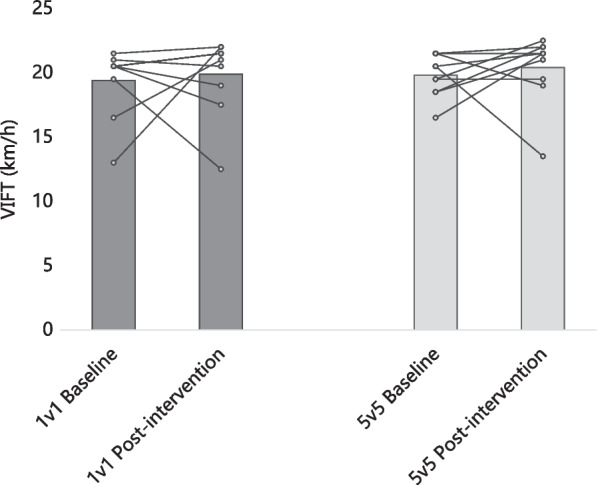
Group average and intra-individual variation of VIFT (final velocity at 30–15 Intermittent Fitness test)

Figure 3 presents the group average and the intra-individual variation of VIFT. VIFT changed 2.6% (19.4 ± 2.6 km/h baseline to 19.9 ± 3.0 km/h post-intervention; *p* = 0.718; d = 0.178) for the 1v1 group and 3.0% (19.8 ± 1.6 km/h baseline to 20.4 ± 2.7 km/h post-intervention; *p* = 0.593; d = 0.274) for the 5v5 group. No significant differences were found between the groups in VIFT at the baseline (2.1% difference; *p* = 0.688; d = 0.182) and post-intervention (2.5%; *p* = 0.697; d = 0.177).

## Discussion

This study tested the effects of two training programs (one program based on extreme sided-games of 1v1 vs. one program based on the medium-sided game of 5v5) on the physical fitness adaptations of youth soccer players. Specifically, it analyzed the effects of the training programs on the 5–0–5 time, CMJ, and VIFT. The extreme-sided games meaningfully beneficiated the vertical jump height and change-of-direction performance of youth soccer players. The extreme-sided games seem more beneficial than medium-sided games for improving these physical abilities while showing that four weeks were enough to impact the players significantly.

A previous study showed a 5.1% improvement in change of direction performance after an SSG training period in a U-17 group [20], which is similar to the current study's findings for both groups (4.8% and 4.2%). Other studies also observed identical improvements in women [21] and recreational subjects [22]. Furthermore, previous studies revealed that small-sided games provide players with higher accelerations and decelerations per minute throughout the bouts [23–26], which eventually explains the positive adaptations reported. Interestingly, even the larger format, where a lower number of accelerations per minute is observed [27], eventually provided players with enough stimuli to generate positive adaptations after the training. Despite that, we cannot firmly state that since we have not monitored this dose–response relationship, thus must be verified in future research. Even considering the hypothesis, it can be speculated that SSGs provide substantial stimuli for change of direction adaptations and seem an innovative training strategy for soccer players.

Concerning VIFT, a previous study reported a 4.1% change in performance after an SSG training period in basketball [28]. An even higher percentage of change in VIFT in another study (8.2%), in which the protocol combined SSGs and HIIT [29]. Both results contradict the current findings as no difference was detected from the pre to post-test. Although VIFT seems dependent on aerobic power [30], the explanation might be related to the settings of small-sided games used in the current study. It has been shown that VIFT is poorly associated with internal load measures [31], which emphasizes that players need to achieve high external load intensities to develop such skills. As players reached the VIFT values closer to 20 km/h in the pre-test, the intensity, in the percentage of VIFT, might not be enough to lead to adaptations. At this point, the first study adopted a basketball task, in which it is common for players to cover the whole playing area (15 m long) to score, as a basketball game requires great precision from players, which discourages long shoots [32]. It has been shown that larger areas are needed to increase high-speed physical responses [32]. Therefore, the number of high-speed actions observed in the current study might be lower than in the previous one, which explains the differences in the results. Besides, the other research that revealed positive adaptations in IFT performance [29] complemented the SSG training with the HIIT sessions. Therefore, HIIT activities might have played a significant role in providing players with high-speed training stimuli, which explains the results obtained. It has been argued that SSGs might offer weak stimuli for high-speed actions due to the small area available [16] required to achieve high speeds. Moreover, the high-intensity actions do not represent those observed in match-play [33]. Therefore, it is suggested that training sessions with SSGs should be complemented with other training activities to allow players to achieve high speed during training.

In general, previous systematic reviews on the influence of the SSG training regimen on CMJ showed positive adaptations related to CMJ performance [34]. A prior study revealed a 1.9% improvement in CMJ performance after an SSG training period composed of one-to-three-a-side SSGs [20], similar to the current research concerning the 1vs1 SSG group (1.7%). However, it is contrary to what was observed in the 5vs5 group. A study with handball players found similar positive adaptations after a training program composed of 3-a-side SSGs [35]. Interestingly, other studies that adopted 4-a-side SSGs during the training period reported no improvements in CMJ [29, 36], similar to the current results in the 5-a-side protocol (medium-sided game). Therefore, it might be argued that smaller formats potentially impact CMJ more prominently than larger ones. At this point, smaller formats are reported to increase players’ involvement compared to larger ones [37]. They are usually associated with duels and tackles in which strength requirements are likely higher than off-the-ball running actions [14]. Therefore, the inherent characteristics of different small-sided games seem to explain the differences in the adaptations during the training period. Finally, the number of specific SSG training sessions in the current study (twice a week), lower than in previous studies [29, 36], can explain the absence of positive adaptations in all the protocols.

This study presents some methodological limitations. One limitation is that no contrast group was analyzed (e.g., a group only performed regular training). Additionally, no dose–response relationship was established. Thus, it is impossible to firmly affirm the recommended dose to promote a given physical adaptation. This research cannot be generalized since it only represents the context of the non-professional youth team. Finally, this research was conducted in the last third of the season, which means that some fatigue-based effects or another contextual factor can influence the final results.

Although their limitations, this study provides exciting pieces of information for practitioners. Intervention studies with small-sided games are welcome to verify whether the acute differences observed when manipulating task constraints lead to different adaptations. The current study confirms this assumption regarding CMJ, as positive adaptations were reported only in the 1vs1 group. As a practical implication, this implies that coaches are encouraged to select tasks following the training session objective to maximize the positive effects of the SSG training. On the other hand, further studies considering (a) more SSG training sessions per week; (b) longer training interventions might be necessary to analyze whether specific SSG characteristics induce different adaptations in VIFT and 5–0–5 time; and (c) testing interactions between extreme and medium sided games aiming to maximize the positive effects of both formats on the physical adaptations of the players.

Moreover, the training regimen in the current study was kept constant over the weeks. Nonetheless, positive adaptations were reported in CMJ and 5–0–5 time. However, there is still a gap in the literature investigating whether systematically increasing the training load would improve positive adaptations. Finally, the current research was conducted in a specific context of non-professional youth players, which should be carefully considered not to generalize the findings for another context (e.g., professionals. Future studies should expand this research to other populations.

## Conclusions

The current research showed that the exposure of youth soccer players to a 1v1 duel twice per week over four weeks could enhance change of direction and vertical jump. However, it does not meaningfully impact the locomotor profile. On the other hand, exposure to the 5v5 format significantly improved change-of-direction, with no considerable differences in a vertical jump or locomotor profile. Duels can be an exciting approach to creating power-related activities with positive transfer to power-related components of physical fitness. Although not possible to generalize the current research, coaches may consider implementing extreme-sided games as part of the training program aiming to ensure a high-intense ecological drill-based stimulus while enhancing physical qualities such as vertical jump or change of direction.

## Supplementary Information

**Additional file 1.** Dataset of the study.

## Data Availability

All data generated or analyzed during this study are included in this published article and its supplementary information files (Additional file 1: Dataset of the study).
